# Successful fenofibrate therapy for severe and persistent hypertriglyceridemia in a boy with cirrhosis and glycerol‐3‐phosphate dehydrogenase 1 deficiency

**DOI:** 10.1002/jmd2.12125

**Published:** 2020-04-30

**Authors:** Lorenza Matarazzo, Valentina Ragnoni, Cristina Malaventura, Alberta Leon, Davide Colavito, Giovanni Battista Vigna, Giovanni Lanza, Aurelio Sonzogni, Giuseppe Maggiore

**Affiliations:** ^1^ Department of Medicine, Surgery, and Health Sciences University of Trieste Trieste Italy; ^2^ Section of Pediatrics, Department of Medical Sciences University of Ferrara Ferrara Italy; ^3^ Research & Innovation Srl (R&I Genetics) Padova Italy; ^4^ Medical Department University of Ferrara Ferrara Italy; ^5^ Department of Medical Sciences, Pathology Unit University of Ferrara Ferrara Italy; ^6^ Department of Pathology ASST Papa Giovanni XXIII Bergamo Italy; ^7^ Gastrointestinal, Liver and Nutrition Unit IRCCS Ospedale Pediatrico Bambino Gesù Rome Italy

**Keywords:** fenofibrate, Glycerol‐3‐phosphate dehydrogenase 1 deficiency, *GPD1* gene, hepatic steatosis, hepatomegaly

## Abstract

Glycerol‐3‐phosphate dehydrogenase 1 deficiency is a rare autosomal recessive disorder caused by mutations in the *GPD1* gene (GPD1; OMIM*138420). Very few cases are reported in literature. It usually manifests in early infancy with transient hypertriglyceridemia, hepatomegaly, steatosis, and fibrosis. We report the case of a 16‐year‐old boy followed since the age of 1 year for hepatomegaly, elevated liver enzymes, and persistent hypertriglyceridemia. Abdominal ultrasound showed diffuse liver echogenicity and liver biopsy disclosed cirrhosis with micro and macrovesicular steatosis. Next‐generation sequencing for metabolic and genetic liver diseases was conducted with the identification of the homozygous mutation c.895G>A in *GPD1* gene resulting in the aminocidic substitution p.G299R. Considering the persistent and progressive increase of plasma triglycerides, fenofibrate treatment was started at 15 years of age allowing triglyceride level reduction in the following 1‐year follow‐up.

## SYNOPSIS

GPD1 deficiency usually manifest in infancy with transient hypertriglyceridemia, elevated liver enzymes, hepatomegaly, and liver steatosis. In some patients, hypertriglyceridemia could be severe and persist overtime. For these patients, fenofibrate represents a possible therapeutic option

## 1 INTRODUCTION

Glycerol‐3‐phosphate dehydrogenase 1 deficiency is a rare autosomic recessive disease caused by mutations in the glycerol‐3‐phosphate dehydrogenase 1 gene (*GPD1*; OMIM*138420), mapped on chromosome 12q12‐q13, which encodes the cytoplasmic NADH/NAD^+^‐ dependent glycerol‐3‐phosphate dehydrogenase 1.^1^ GPD1 has a pivotal role in both carbohydrate and lipid metabolism. *GPD1* mutations were first described in 2012 in 10 Israeli Arab patients presenting with early onset hepatomegaly, hepatic steatosis, and hypertriglyceridemia in the first year of life.^2^ To date, only 17 patients were reported in five articles showing transient hypertriglyceridemia in the majority of cases.^2^, ^3^, ^4^, ^5^, ^6^

Herein we describe the case of a boy affected by GPD1 deficiency who presented severe and persistent hypertriglyceridemia, successfully treated with fenofibrate.

## 2 CASE REPORT

A 16‐year‐old adopted Russian boy presented at 1 year of age with hepatomegaly and mild increase of liver enzymes (alanine aminotransferase, ALT 1.5xULN, gamma‐glutamyl transferase, GGT, 2xULN). Laboratory tests revealed high plasma triglycerides (274 mg/dL), normal cholesterol (166 mg/dL), HDL, and LDL levels.^7^ Biliary acids were slightly increased 16.6 μmol/L (normal value, nv <10). Lactic acid was 4.40 mmol/L (nv < 2.47). Bilirubin, alpha‐fetoprotein, synthetic liver function (coagulation and albumin), glucose, and renal function were within the normal range. Clinical examination showed marked hepatomegaly without splenomegaly, normal growth, and psychomotor development. Body mass index (BMI) at 3.8 years of age was 14.7 kg/m^2^ (10‐25° percentile).^8^

Laboratory investigations (including blood and urine routine tests, blood gases, uric acid, immunoglobulin levels, thyroid function, plasma amino acids profile, urine organic acids, creatine phosphokinase, viral serology, ceruloplasmin, and acid lipase) and an echocardiogram were all within normal limits. Moreover, a 14‐hours fasting test revealed no hypoglycemia. Liver ultrasound highlighted hepatomegaly with diffuse echogenicity.

**FIGURE 1:**
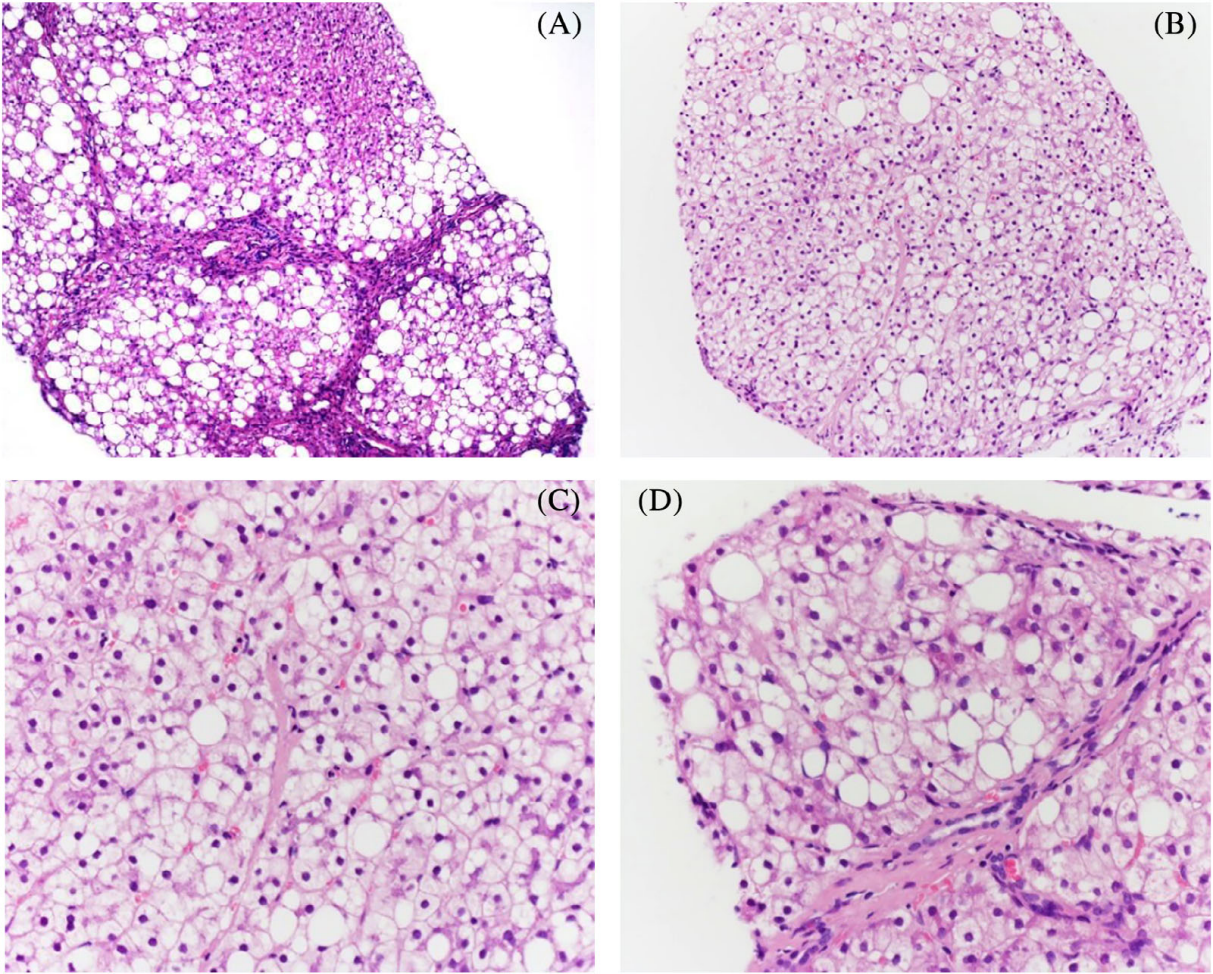
Liver tissue showing diffuse nodular transformation of parenchyma without significant inflammatory infiltrate (H&E ×40), A; histological details of liver parenchymal damage demonstrating extensive macrovesicular and microvesicular steatosis; nonsteatotic liver cells were characterized by cytoplasmatic microvacuolization and enlargement (H&E, ×100), B,C; follow‐up liver biopsy showed unchanged histological features (D) (H&E ×40)

A first liver biopsy, performed at 3 years of age, showed an incomplete diffuse cirrhotic pattern with mild and focal mixed inflammatory infiltrate. The main histological features were liver cells alterations with severe large and small droplets steatosis, highly suggestive for an unspecified metabolic disease (Figure 1A‐C).

**FIGURE 2:**
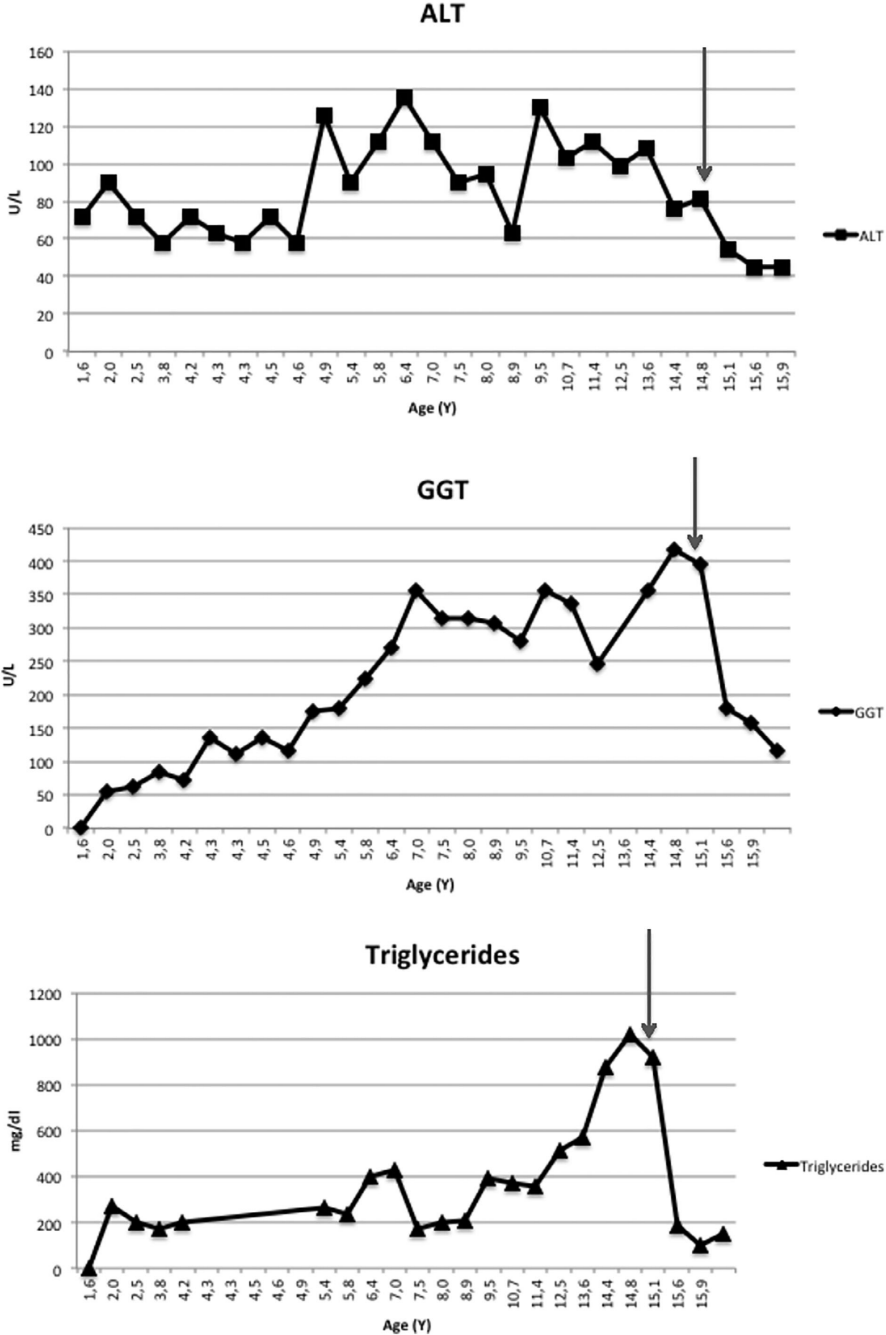
ALT, GGT, and triglyceride levels during follow‐up. Gray lines indicate fenofibrate onset

During the following years a persistent elevation of liver enzymes was recorded (ALT 3xULN, GGT 9xULN) while plasma total cholesterol and triglycerides peaked at 315 and 1017 mg/dL, respectively (Figure 2). BMI value and patient's parameters at each follow‐up visit were reported (Table 1). At 15 years of age, a second liver biopsy confirmed the histological changes of the previous one, but findings were more pronounced (Figure 1D). A clinical examination at 16 years of age revealed hard hepatomegaly, normal spleen size, and no skin or tendineous xanthomas. BMI value was 24.8 kg/m^2^ (75‐90°percentile).^8^ Repeated abdominal ultrasound investigation confirmed hepatomegaly, left lobe hypertrophy, and increased echogenicity without signs of portal hypertension. Echocardiogram and supra‐aortic trunks ultrasound tested normal. During follow‐up liver elastometry (Fibroscan) was performed.

**TABLE 1:** Patient's parameters during follow up

Follow‐up	TG	TC	HDL	LDL	ALT	AST	GGT	BMI
Age (y,m)	mg/dL	mg/dL	mg/dL	mg/dL	UI/L	UI/L	UI/L	kg/m^2^
2	203	189	46	‐	90	103	63	‐
2,5	174	196	‐	‐	72	76	85	‐
3,8	197	195	‐	‐	58	67	72	14,7 (10‐25° pc)
4,9	266	193	63	102	126	198	180	16,6 (50‐75°pc)
5,4	239	239	62	129	90	135	225	16,5 (50‐75°pc)
5,8	399	251	65	‐	112	247	270	‐
6,4	430	255	54	‐	135	310	355	16,8 (50‐75°pc)
7	174	261	77	‐	112	180	315	16,6 (50‐75°pc)
8	206	265	81	142	94	153	306	17,3 (50‐75°pc)
8,9	396	234	62	92	63	121	279	17,6 (50‐75°pc)
9,5	369	274	78	122	130	252	355	18,3 (50‐75°pc)
10,7	354	257	54	131	103	189	337	17,7 (25‐50°pc)
11,4	515	271	58	157	112	211	247	19,4 (50‐75°pc)
12,5	571	251	‐	‐	99	139	‐	21,4 (50‐75°pc)
13,6	878	299	‐	‐	108	135	355	21,9 (50‐75°pc)
14,4	1017	315	‐	‐	76	135	418	23 (50‐75°pc)
15,1	183	217	94	‐	54	103	180	24,5 (75‐90°pc)
15,6	103	189	91	‐	45	81	157	24,8 (75‐90°pc)
15,9	147	166	71	‐	45	45	117	24,8 (75‐90°pc)

*Note:* Bold values indicate data following fenofibrate onset. BMI percentile from reference 8.

Abbreviations: pc, percentile; TC, total cholesterol; TG, triglycerides.

Next‐generation sequencing (NGS) for metabolic and genetic liver diseases identified a novel homozygous mutation in the *GPD1* gene, c.895G>A resulting in the amminocidic substitution p.G299R.

Due to the persistent and progressive increase of triglycerides values, after consultation with a lipidologist, an off label therapy with fenofibrate was started at 15 years of age at the dosage of 145 mg every other day. The local ethical committee approved this treatment. During a further 15‐months follow‐up, fenofibrate led to ALT normalization and to a marked reduction of triglyceride plasma levels (last value 147 mg/dL). GGT and total cholesterol also decreased to 2.5xULN and 166 mg/dL, respectively. (Table 1, Figure 2). The elastometry values before starting fenofibrate and after 1 year of therapy were respectively 11.5 and 10.6 kPa, compatible with F2 fibrosis. No drug‐related side effects were observed.

## 3 GENETIC ANALYSIS

Whole blood (3 mL) was collected for exome analysis after obtaining informed consent. DNA was extracted using the Qiagen BioRobot DNA extraction kit (Qiagen Benelux B.V., Venlo, the Netherlands) according to the manufacturer's instructions and quantified using Nanodrop spectral analysis (Thermo Fischer Scientific, Inc., Waltham, Massachusetts). DNA fragmentation and degradation were evaluated by standard agarose gel electrophoresis (100 V, 30 minutes, 1.5% agarose gel in Tris‐borate‐EDTA buffer). DNA Library preparation and whole exon enrichment were performed employing Agilent All Exon V.6 kit (Agilent Technologies, Inc., Santa Clara, California). Library sequences were obtained using the HiSeq2500 Illumina Sequencer (125‐bp paired end sequence mode). Bioinformatics analysis included the following: NGS reads mapping to whole genomes using the Burrows‐Wheeler Alignment tool with default parameters, polymerase chain reaction (PCR) duplicate removal using Picard (http://picard.sourceforge.net), single nucleotide polymorphisms and indel calling using the Genome Analysis Toolkit (GATK) UnifiedGenotyper, variant annotation using snpEff (http://snpeff.sourceforge.net) and false positive variant filtration using the GATK VariantFiltration module. Exome sequencing data and reads alignment analysis were checked for coverage depth and alignment quality employing Bedtools software package. Variant classification was performed in accordance with the guidelines from the American College of Medical Genetics and Genomics.

Phenotype driven analysis coupled with the employment of in silico multigene panels specific for liver diseases and metabolic diseases was used to filter, select and interprets genetic variants obtained following exome sequencing. The presence of significant variants were confirmed by Sanger sequencing.

NGS sequencing analysis highlighted the presence of the novel homozygous mutation c.895G>A in the *GPD1* gene resulting in the amminocidic substitution p.G299R. The mutation here found is absent from allele frequency databases (ExAC, EVS, and GnomeAD), Bioinformatics predictors indicate that the Glycine in position 299 is highly conserved through high vertebrates (PhyloP Vertebrates = 5,51/6,42; PhyloP‐Primate = 0,46/0,65) and the missense change Glicine to Arginine is likely to be deleterious (Polyphen2 = 0,921/1,00; SIFT = 0,00/0,00; CADD‐Phred = 28).

## 4 DISCUSSION

Hypertrigliceridemia may have primary (genetic), or secondary (obesity, metabolic syndrome, diabetes, hypothyroidism, liver disease, alcohol use) causes and is often multifactorial.^9^

Genetic causes include inborn errors of metabolism related to defects in triglyceride synthesis, storage, or degradation. Glycerol‐3‐phosphate (G3P) plays a key role in triglyceride synthesis. In adipose tissue, G3P is mainly derived from dihydroxyacetone phosphate (DHAP), which represents a by‐product of glycolysis and glyceroneogenesis. Two glycerol phosphate dehydrogenase enzymes encoded by the genes *GPD1* (cytoplasmic GPDH, GPDH‐C) and *GPD2* (mitochondrial GPDH, GPDH‐M) mediate the transport of reducing equivalents from the cytosol to mitochondria. The GPDH‐C reaction is reversible and uses NADH/NAD^+^ as cofactor; GPDH‐M is irreversible and uses FADH as cofactor.^1^

Deficiency of GPDH‐C causes glycerol‐3‐phosphate dehydrogenase 1 deficiency, a rare autosomal recessive disorder, which has been genetically confirmed in only 17 patients up to now.^2^, ^3^, ^4^, ^5^, ^6^ The disease was at first described in humans by Basel‐Vanagaite et al in 2012.^2^ Its main features include moderate to severe hypertriglyceridemia, elevated liver enzymes, hepatomegaly, liver steatosis and fibrosis appearing in early childhood. Differential diagnosis includes NAFLD/NASH, glycogen storage disease, citrin deficiency, lysosomial acid lipase deficiency, and lipidosis.^4^, ^6^

In their study, Basel‐Vanagaite et al^2^ identified 10 affected members from four Israeli Arab families, both consanguineous and nonconsanguineous. In the first year of life affected individuals disclosed vomiting and failure to thrive but half cases were asymptomatic. Patients had moderate to severe hepatomegaly and in three cases splenomegaly. Fasting hypertriglyceridemia was consistently present ranging from mild to severe (between 258 and 6244 mg/dL), as well as elevated transaminases and GGT. Cholesterol levels were normal at disease onset (except in one patient) and moderately increased in nine subjects during follow‐up, remained within normal value in all but three. All patients had fatty liver at the abdominal ultrasound. At their last evaluation, transaminases were still elevated in most cases, while triglyceride decreased in eight, even if higher than 200 mg/dL in all but one. Two patients underwent liver biopsies, which demonstrated marked macro and microsteatosis with fibrosis, septal formation, and mild inflammatory infiltrates. The analysis of the genetic defects showed homozygosity for a splicing mutation c.361‐1G>C, in *GPD1*. Heterozygotes did not manifest hepatic involvement or increased triglyceride levels.

In 2014, Joshi et al^3^ described a Caucasian child with hepatomegaly, fatty liver, and severe hypertriglyceridemia. The patient presented with failure to thrive, vomiting, and early hepatomegaly with an increase in plasma transaminases, GGT, triglycerides (839 mg/dL) and cholesterol (197 mg/dL). Liver ultrasound revealed hepatomegaly with hyperechogenicity, and liver biopsy showed marked micro‐macrovesicular steatosis. At last follow‐up visit at 1.5 years of age plasma triglycerides slightly decreased at 536 mg/dL. This patient inherited a *GPD1* deletion from her father and a missense mutation p.R229Q from her mother; this was the first report describing a patient carrying mutations in both alleles of the *GPD1* gene (compound heterozygote).

Later in 2016, Dionisi‐Vici et al^4^ reported four additional patients from three families who showed some phenotypical differences. In particular, a patient experienced a significant and recurrent fasting hypoglycemia associated with elevated liver enzymes, hepatomegaly, and mild hypertriglyceridemia. Moreover, another patient showed severe liver disease with signs of intrahepatic cholestasis, kidney involvement and organic aciduria. Whole exome sequencing revealed biallelic mutations in the *GPD1* gene, including two novel variants (c.806G>A, p.Arg269Gln and C.640T>C, p.Cys214Arg) and a previously reported splice site variant (c.361‐1G>C).

In 2017 Li et al^5^ reported of a Chinese boy with a somewhat different clinical phenotype from previous cases, characterized by obesity, insulin resistance, fatty liver, dermal abnormalities (facial acne, acanthosis nigricans, and hirsutism), short stature, elevated dehydroepiandrosterone sulfate and lipoprotein‐α levels. His liver exams, triglyceride, and cholesterol were normal, but abdominal ultrasound showed fatty liver. Targeted‐NGS revealed that the patient was a compound heterozygote for c.220‐2A>G and c.820G>A mutation in the *GPD1* gene.

In 2018, a Chinese girl was described by Li et al^6^ with elevated ALT and GGT detected at the age of 3 months. Abdominal ultrasound revealed hepatomegaly and fasting hypertriglyceridemia was noted at presentation (388 mg/dL) and last evaluation (370 mg/dL), with normal cholesterol. Liver biopsy performed at 6.5 months of age showed severe hepatic steatosis, mild lymphocytic inflammatory infiltrates in the portal tracts, and mild portal fibrosis. A homozygous nonsense mutation c.523C>T, p. (p.Q175*) in *GPD1* was identified.

Basel‐Vanagaite et al^2^ suggested that hypertriglyceridemia and fatty liver disease in their patients could be related to an increased hepatic synthesis of triglycerides, decreased export from the liver, increased uptake of fatty acids into the liver and reduced beta oxidation. They confirmed in HepG2 cells that pathogenetic mutations of *GPD1* may alter several steps of triglyceride metabolism.

Among all previous 17 published patients carrying mutations in *GPD1*, there was a high prevalence of homozygous condition (15 patients), as we found in our case, while only two children were compound heterozygotes.

The patient we present in this paper has features of GPD1 deficiency such as early disease onset, increase of ALT and GGT levels, fatty liver disease, and fibrosis. Hypertriglyceridemia increased over time reaching the value of 1017 mg/dL, while in previous reports only about one fourth of patients did not improve triglyceride levels on follow‐up, even if mild hypertriglyceridemia may frequently persist (> 200 mg/dL, with a maximum value of 536 mg/dL). He showed liver cirrhosis at biopsy when he was 3 years old and his triglycerides were only slightly increased. After 12 years a new biopsy confirmed the same results, but in this latter case triglyceride plasma levels were markedly elevated. Our patient did not experience coronary disease or pancreatitis. However, given the very high plasma lipid levels, after multidisciplinary discussion we proposed an off label fenofibrate therapy that was successfully started after ethics committee approval.

The exact effects of fenofibrate are not entirely understood. Still, it acts by the activation of the nuclear transcription factor peroxisome proliferator‐activated receptor‐α (PPAR α) which regulate lipid metabolism. Thus, fenofibrate lowered the concentration of triglyceride by reducing the synthesis and increasing the hydrolysis of triglycerides‐rich lipoproteins. Fenofibrate leads to a decrease of serum triglyceride by 20% to 50%, proportionally to the baseline triglyceride levels. Fenofibrate also increased HDL‐C and has variable effects on LDL‐C levels depending on the type of dylipidemia.^10^

To date, little has been published about the use of agents for treating hypertriglyceridemia in children, while efficacy and safety data are lacking. There are no Food and Drug Administration (FDA) approved triglyceride‐lowering drugs for use under 18 years of age, and pediatric approach to lipid disorders must be prudent.^9^, ^11^ The Summary Report of the Expert Panel on Integrated Guidelines for Cardiovascular Health and Risk Reduction in Children and Adolescents^7^ states that people aged 10 to 21 years, with lipid abnormalities (LDL cholesterol level of ≥250 mg/dL and/or triglyceride level of >500 mg/dL) should be managed for 3 to 6 months with diet modifications. Children at high risk, e.g. with severe primary dyslipidemia, who cannot reach lipid targets with diet alone should concurrently be considered for medication therapy, after consultation with a lipid specialist. Fibrates (in particular fenofibrate, bezafibrate) represent the first‐line therapy to reduce pancreatitis risk when triglyceride concentration is greater than 500 mg/dL. They can rarely cause myopathy and are contraindicated in patients with severe renal impairment or gallbladder disease.^9^

Rarely fenofibrate and other fibrates may be responsible for mild and temporary serum aminotransferase elevations and even occasional instances of acute liver injury. For this reason we undertook a prudent therapy, with frequent control of plasma liver enzymes and we were quite satisfied for their normalization. It is concevable that treatment, as is the case for all lipid‐lowering agents, should be continued indefinitely, but transience of hypertriglyceridemia in other cases of this condition might induce to tapering‐off this drug after only a few years.^12^

The natural history of glycerol‐3‐phosphate dehydrogenase 1 deficiency is not clearly defined. Affected individuals seem to exhibit a rather good medium term prognosis even in the presence of liver fibrosis arisen during early infancy. In addition no clinical evidence of coronary heart disease or episodes of pancreatitis has been reported, neither in the oldest patients^2^, ^4^; however, no conclusions can be made about risk in adult age or after long‐term follow‐up. Considering these aspects, as also reported in the previous articles, liver transplantation is usually not recommended. However, since the metabolic indication for liver transplantation are increasing and considering the persistent chronic liver damage in our patient, the possibility of liver transplantation in the future could not be excluded.

In conclusion, GPD1 deficiency should be suspected in case of early occurrence of hypertriglyceridemia, elevated liver enzymes, hepatomegaly, liver steatosis, and fibrosis in infancy. Even if long follow up data are still not available, the medium‐term prognosis does not seem poor. Hypertriglyceridemia may become a relevant clinical problem in some cases and fenofibrate represents a possible therapeutic option for these patients. A lipid‐clinic evaluation and a close follow‐up are recommended.

## CONFLICT OF INTEREST

L. M., V. R., C. M., A. L., D. C., G. B. V., G. L., A. S., and G. M. declare that they have no conflict of interest.

## AUTHOR CONTRIBUTIONS

Lorenza Matarazzo: conception and design, collection and assembly of data, data analysis and interpretation, manuscript writing, final approval of manuscript. Valentina Ragnoni: collection and assembly of data, data analysis and interpretation, manuscript writing, final approval of manuscript. Cristina Malaventura: manuscript writing, final approval of manuscript. Alberta Leon: manuscript writing, final approval of manuscript. Davide Colavito: manuscript writing, final approval of manuscript. Giovanni Battista Vigna: manuscript writing, final approval of manuscript. Giovanni Lanza: manuscript writing, final approval of manuscript. Aurelio Sonzogni: manuscript writing, final approval of manuscript. Giuseppe Maggiore: conception and design, data analysis and interpretation, manuscript writing, final approval of manuscript.

## ETHICS STATEMENT

All procedures followed were in accordance with the ethical standards of the responsible committee on human experimentation (institutional and national) and with the Helsinki Declaration of 1975, as revised in 2000 (5). Informed consent was obtained from all patients for being included in the study. This article does not contain any studies with human or animal subjects performed by the any of the authors.
